# Cell-bound lipases from *Burkholderia* sp. ZYB002: gene sequence analysis, expression, enzymatic characterization, and 3D structural model

**DOI:** 10.1186/s12896-016-0269-6

**Published:** 2016-05-03

**Authors:** Zhengyu Shu, Hong Lin, Shaolei Shi, Xiangduo Mu, Yanru Liu, Jianzhong Huang

**Affiliations:** National & Local United Engineering Research Center of Industrial Microbiology and Fermentation Technology, Ministry of Education, Fujian Normal University, Fuzhou, 350117 China; Engineering Research Center of Industrial Microbiology, Ministry of Education, Fujian Normal University, Fuzhou, 350117 China; College of Life Sciences, Fujian Normal University (Qishan campus), Fuzhou, 350117 China

**Keywords:** *Burkholderia* sp. ZYB002, Cell-bound lipase, Lipase LipC24, Lipase LipA

## Abstract

**Background:**

The whole-cell lipase from *Burkholderia cepacia* has been used as a biocatalyst in organic synthesis. However, there is no report in the literature on the component or the gene sequence of the cell-bound lipase from this species. Qualitative analysis of the cell-bound lipase would help to illuminate the regulation mechanism of gene expression and further improve the yield of the cell-bound lipase by gene engineering.

**Results:**

Three predictive cell-bound lipases, *lipA*, *lipC21* and *lipC24*, from *Burkholderia* sp. ZYB002 were cloned and expressed in *E. coli*. Both LipA and LipC24 displayed the lipase activity. LipC24 was a novel mesophilic enzyme and displayed preference for medium-chain-length acyl groups (C10-C14). The 3D structural model of LipC24 revealed the open Y-type active site. LipA displayed 96 % amino acid sequence identity with the known extracellular lipase. *lipA*-inactivation and *lipC24*-inactivation decreased the total cell-bound lipase activity of *Burkholderia* sp. ZYB002 by 42 % and 14 %, respectively.

**Conclusions:**

The cell-bound lipase activity from *Burkholderia* sp. ZYB002 originated from a multi-enzyme mixture with LipA as the main component. LipC24 was a novel lipase and displayed different enzymatic characteristics and structural model with LipA. Besides LipA and LipC24, other type of the cell-bound lipases (or esterases) should exist.

**Electronic supplementary material:**

The online version of this article (doi:10.1186/s12896-016-0269-6) contains supplementary material, which is available to authorized users.

## Background

Microbial lipase (triacylglycerol lipase, EC 3.1.1.3) catalyze hydrolysis of the long chain triglycerides, or the reverse reaction. Besides hydrolysis activity, lipases also displayed alcoholysis, aminolysis, interesterification, and esterification activity, etc. with rigorous regioselectivity, stereoselectivity, and chemoselectivity [1]. As one kind of non-aqueous enzymes, lipases kept high catalysis efficiency in organic solvent systems or micro-aqueous systems, and were widely used in many industrial fields [2].

Microbial strains can produce multiple types of lipases. *Candida rugosa* produced more than five types of lipase isoenzymes (Lip1-Lip5), which shared high sequence identity, but displayed significantly different enzymatic characteristics [3]. Different types of lipases produced by a specific microbial strain always were distributed to different cell compartments, respectively. Lipase LipA from *Pseudomonas aeruginosa* either was secreted into the culture medium, or interacted with the polysaccharide alginate and then anchored on the cell surface [4–6], while Esterase EstA from *P. aeruginosa* was located in the outer membrane [7, 8].

Cell-bound lipase could be directly used as a whole cell biocatalyst. Compared with the extracellular enzyme, whole cell biocatalysts displayed many advantages, including high stability in the long-term, inexpensive preparation, independence of the exogenous co-factor for redox reaction, etc. [9]. In previous research, *Burkholderia* sp. ZYB002 produced both extracellular lipase and cell-bound lipase [10, 11]. The cell-bound lipase from *Burkholderia cepacia* displayed excellent interesterification activity for biodiesel production and highly enantioselective hydrolysis activity for L-menthol synthesis [12, 13]. However, there wasn’t any report on the type or the gene sequence of the cell-bound lipase from *B. cepacia*.

In this article, three predictive cell-bound lipase genes from *Burkholderia* sp. ZYB002, *lipA*, *lipC21* and *lipC24*, were cloned and expressed in *E. coli*, respectively. Furthermore, the component of the cell-bound lipase from *Burkholderia* sp. ZYB002 was analyzed.

## Methods

### Bacterial strains and plasmids

The bacterial strains and plasmids used in this study are listed in Table 1. Briefly, *E. coli* DH5α was used as the host strain for plasmid amplification, and *E. coli* BL21(DE3) and *E. coli* Origami2 (DE3) were used as the expression host strain for three lipase genes, *lipA*, *lipC21* and *lipC24*, respectively. *Burkholderia* sp. ZYB002 was the lipase-producing strain, which was isolated and identified in our lab [14]. Antibiotics were added as required to the final concentrations of 60 μg/mL ampicillin, 35 μg/mL chloramphenicol, 50 μg/mL kanamycin, 100 μg/mL trimethoprim, 50 μg/mL gentamicin.

**Table 1:** Strains and plasmids used in the current study

	Description	Source
Strains		
*Burkholderis* sp. ZYB002	Wild-type, lipase-producing strain with multiple antibiotic resistance	Shu et al., 2009 [14]
*Burkholderis* sp. ZYB002 -Δ*lipA*	*lipA*-inactivation mutant strain derived from *Burkholderis* sp. ZYB002; Tmp^r^ and *lipA*::gfp	This study
*Burkholderis* sp. ZYB002 -Δ*lipC24*	*lipC24*-inactivation mutant strain derived from *Burkholderis* sp. ZYB002; Tmp^r^ and *lipC24*::gfp	This study
*E. coli* DH5α	*fhuA2 lac(del)U169 phoA glnV44 Φ80’ lacZ(del)M15 gyrA96 recA1 relA1 endA1 thi-1 hsdR17*	TAKARA
*E. coli* BL21(DE3)	Expression host strain for *lipA* and *lipC21*	Novagen
*E. coli* Origami2 (DE3)	Expression host strain for *lipC24*	Novagen
Plasmids		
pMD18T-*lipAB*	pMD18T containing the PCR-amplified *lipA* and *lipB*	This study
pMD18T-*lipC21*	pMD18T containing the PCR-amplified *lipC21*	This study
pMD18T-*lipC24*	pMD18T containing the PCR-amplified *lipC24*	This study
pEDSF-*lipB*	pACYCDuet-1 with insertion of *lipB* at MCS2	This study
pEDSF-*lipB*-*lipA*	pEDSF-*lipB* with insertion of *lipA* at MCS1	This study
pEDSF-*lipC21*	pET28a with insertion of *lipC21* at MCS	This study
pEDSF-*lipB*-*lipC24*	pEDSI-*lipB* with insertion of *lipC24* at MCS1	This study
pGro7	Expression plasmid containing *groES-groEL* gene, *ara*B promoter	TAKARA
pBBR1TP	Broad host range cloning vector with the trimethoprim resistance gene	Yingrun Bio. Inc.
pJQ200SK	Suicide vector with gentamicin resistance gene	Yingrun Bio. Inc.
pRK2013	The helper plasmid with RK2 transfer genes and kanamycin resistance gene	Yingrun Bio. Inc.
pEGFP-N1	Expression vector with *gfp* gene and kanamycin resistance gene	Clontech.
pBCMB-S1	pJQ200SK containing the PCR-amplified *tmp* gene from pBBR1TP	This study
pBCMB-S2	pBCMB-S1 containing the PCR-amplified *lipA* gene fragment (*lipA* ^,^)	This study
pBCMB-S3	pBCMB-S2 containing the PCR-amplified *gfp* gene from pEGFP-N1	This study
pBCMB-S4	pBCMB-S1 containing the PCR-amplified *lipC24* gene fragment (*lipC24* ^,^)	This study
pBCMB-S5	pBCMB-S4 containing the PCR-amplified *gfp* gene from pEGFP-N1	This study

The cloning plasmid pMD18T-*lipAB*, pMD18T-*lipC21*, and pMD18T-*lipC24*, harbored the full length lipase gene of *lipA* and its chaperonin gene *lipB*, *lipC21* and *lipC24*, respectively. The expression plasmid pEDSF-*lipB-lipA*, pEDSF-*lipC21*, and pEDSF-*lipB-lipC24*, harbored the coding region for the mature LipA (lipase A) and its chaperonin LipB (the lipase-specific foldase), LipC21 (lipase C21), and LipC24 (lipase C24)/LipB, respectively.

### Chemicals and biochemistry reagents

High-fidelity DNA polymerases, restriction enzymes, T_4_-DNA ligases, PCR purification kits, the DNA Gel-Extraction Kits, DNA markers, and protein markers etc. were purchased from Takara Biotechnology Co. Ltd (Dalian, China). Primers synthesis and DNA sequencing was completed by Sangon Biotechnology Co. Ltd (Shanghai, Beijing). All antibiotics were purchased from Beijing dingguo changsheng biotechnology Co. Ltd (Beijing, China). Various 4-nitrophenyl fatty acid esters, triolein, oleic acid, 1, 3-diolein, 1, 2-diolein and 1-monoolein were purchased from Sigma-Aldrich. Silica gel GF254 was purchased from Haiyang Chemical Co. Ltd (Qingdao, China). Olive oil, *n*-hexane, chloroform and acetone were of analytical grade and purchased from Sinopharm Chemical reagent Co. Ltd (China).

### Gene cloning and sequence alignment of *lipA, lipC21* and *lipC24*

The full lengths of three different lipase genes, *lipA*/*lipB*, *lipC21* and *lipC24* were amplified by PCR using the genomic DNA from *Burkholderia* sp. ZYB002 as the templates. The primer pairs for PCR were listed in the Table 2. All PCR conditions and PCR procedures used in this research were given in the Additional file 1. PCR products were ligated into pMD18-T simple vector to construct the cloning plasmid pMD18T-*lipAB*, pMD18T-*lipC21*, and pMD18T-*lipC24*, respectively. The three lipase genes were sequenced in full length.

**Table 2:** Oligonucleotide primers used in the current study^a^

Primers	Oligonucleotide sequence (5’ to 3’)	Annealing temperature (°C)	PCR products
lipACF	AAGGATCCTCGGCGTCGACAACGTGCTGAACAAG	52	Full length of *lipA* and the corresponding chaperonin *lipB*
lipACR	CGAAAGCTTCGCCAACACCATCGAGCAACATCTG		
lipC21CF	TCGATGGCTTGGGTGACGGACA	59	Full length of *lipC21*
lipC21CR	CGAAGTTGGCTGGCACTCTTTGGC		
lipC24CF	CTAGTGCAGCGTCTCGGGCGCGA	62	Full length of *lipC24*
lipC24CR	CACCATGTCCTCCAGACGTTTCATGATGG		
lipBEF	TATAGATCTCCCGCCGTCGCTCGCCGGCTCCAG	75	The coding region for the truncated LipB with deletion of N-terminal 70-amino acid residue.
lipBER	CTTCTCGAGCTGCATGCTGCCGGCCCCGCG		
lipAEF	TATGGATCCGGCCGATGGCTACGCGGCGACGC	73	The coding region for the mature LipA
lipAER	CTTAAGCTTTTACACGCCCGCCAGCTTCAG		
lipC21EF	CGCGGATCCGCTTCGCCCGGCCGCGTTCCC	60	The coding region for the mature LipC21
lipC21ER	CCCAAGCTTGCCGCGACACGGCCTGCTGCGC		
lipC24EF	CGCGGATCCGGCGCACCGGCCGTGTCCGA	60	The coding region for the mature LipC24
lipC24ER	CCCAAGCTTGGTGCAGCGTCTCGGGCGCGAG		
lipC24MF	GCTAT**GCA**GGCGGCGCGATCGCGAC	60	pEDSF-*lipB*-*lipC24-Ser* ^*179*^ *Ala*
lipC24MR	GCCGCC**TGC**ATAGCCGATCATCGCG		
tmpF	CTTAGATCTCACGAACCCAGTTGACATAAG	54	Full length of the trimethoprim resistance gene
tmpR	CTTAGATCTTTAGGCCACACGTTCAAG		
lipAIF	CTTGGATCCCGAGTATTGGTACGGCATCCAG	53	*lip*A gene fragment (named as *lip*A^’^)
lipAIR	CTTCTCGAGTTACACGCCCGCCAGCTTCAGC		
gfpF-lipA	CTTCTGCAGATGGTGAGCAAGGGCGAGGA	54	Full length of the *gfp* gene (construction pBCMB-S3)
gfpR-lipA	CTTCTGCAGTTACTTGTACAGCTCGTCCATG		
LipC24IF	TGCTCTAGAAATACGGGATGACCACGCTTGAT	66	*Lip*C24 gene fragment (named as *lip*C24^’^)
LipC24IR	CTTGGGCCCCGTTGAAACGGTCGTAGAGCCAC		
gfpF-lipC24	GGAATTCCATATGATGGTGAGCAAGGGCGAGGA	60	Full length of the *gfp* gene (construction pBCMB-S5)
gfpR-lipC24	GGAATTCCATTTACTTGTACAGCTCGTCCATG		

^a^Underlined nucleotides: restriction endonuclease site; Bold nucleotides: the mutated sites

The nucleotide sequences of *lipA/lipB*, *lipC21* and *lipC24* have been deposited in the GenBank database. To construct the phylogenetic tree, the deduced amino acid sequences of LipA, LipC21 and LipC24, were submitted to BLAST at the NCBI web site, respectively. The retrieved-sequences displaying over 30 % sequence identity to LipA, LipC21 or LipC24 were selected and then aligned using BioEdit editor (Version 7.0.1). The phylogenetic tree was constructed using the software MEGA4.

### Construction of the expression plasmids for *lipA, lipC21* and *lipC24*

Plasmid pACYCDuet-1 was selected to functionally co-express *lipA* with *lipB* or *lipC24* with *lipB*, respectively. Plasmid pET28a was selected to functionally express *lipC21*. Primer pairs used for PCR amplification of *lipB*, *lipA*, *lipC21* and *lipC24* fragments were listed in the Table 2. The PCR products, plasmid pET28a, plasmid pACYCDuet-1 and plasmid pEDSF-*lipB* (Table 1) were double digested by the restriction endonuclease, followed by the ligation reaction to yield the expression plasmid pEDSF-*lipB*-*lipA*, pEDSF-*lipC21* and pEDSF-*lipB*-*lipC24*, respectively (Table 1).

### Expression of *lipA, lipC21* and *lipC24* in *E. coli*

*E. coli* BL21(DE3) was used as the expression host strain for pEDSF-*lipB*-*lipA* and pEDSF-*lipC21. E. coli* Origami2(DE3) was selected as the expression host strain for pEDSF-*lipB*-*lipC24*. Chaperone plasmid pGro7 was co-transformed with plasmid pEDSF-*lipC21* into *E. coli* BL21(DE3).

Same induction condition was adopted for *E. coli* BL21(DE3)-pEDSF-*lipB*-*lipA* and *E. coli* Origami2(DE3)-pEDSF-*lipB*-*lipC24*. When the cell density (OD_600_) reached 0.6–0.9, IPTG was added to the culture medium to the final concentration of 1 mmol/L. Induction culture was lasted for 16 h at 25 °C and then the cells were collected by centrifugation.

Expression of *E. coli* BL21(DE3)-pEDSF-*lipC21*/pGro7 was induced by IPTG and L-arabinose, respectively, as described by Pérez et al. [15]. In brief, 0.5 mg/mL L-arabinose was initially added into the culture medium to induce expression of the chaperone gene of *groES-groEL*. IPTG (1 mmol/L final concentration) was not added into culture medium until the cell density (OD_600_) reached 0.6. Induction incubation of IPTG was lasted for 16 h at 25 °C and the cells were then collected by centrifugation.

### Purification of LipA, LipC21, and LipC24

*E. coli* cells were lysed using sonication and the supernatant was collected by centrifugation. The recombinant protein carrying a (His)_6_-tag was purified from the supernatant using immobilized metal-affinity chromatography (HisTrap HP, 1 mL, GE Healthcare) followed by anion exchange chromatography (HiTrap DEAE F. F., 1 mL, GE Healthcare). Before loaded onto the HisTrap chromatography column, the supernatant was incubated with 2 mmol/L ATP for 10 min at 37 °C to dissociate the recombinant protein/chaperone complex.

The loading buffer for affinity chromatography column consisted of the following components, 20 mmol/L Na_2_HPO_4_-NaH_2_PO_4_ buffer (pH7.5),20 mmol/L imidazole,500 mmol/L NaCl. The recombinant protein was eluted using a linear concentration gradient from 20 mmol/L to 1 mol/L imidazole in the same buffer, and the active fractions (or the target fractions identified by anti-His Western-blot) were pooled and dialyzed against 50 mmol/L Tris–HCl buffer (pH7.5). The desalted protein solution was loaded on anion exchange chromatography column using 50 mmol/L Tris–HCl buffer (pH7.5) and eluted using a linear concentration gradient from 0 mol/L to 1 mol/L NaCl in the same buffer.

The homogeneity of the purified protein was determined by SDS-PAGE on a 12 % separating gel in the presence of 0.1 % SDS. The protein concentration was analyzed using the Bradford method, with bovine serum albumin as standard.

### Biochemical characterization of LipC24

Temperature optimum and temperature stability The optimal temperature was determined by incubating the standard reaction mixture at different temperatures ranging from 30 °C to 60 °C, and the maximum lipase activity was considered 100 %. To analyze temperature stability, the LipC24 preparation was incubated at 40 °C and aliquots were continuously taken at 3-min interval to assay the residual activity. Inactivation process of LipC24 preparation was continued until 80 % of the activity was lost. Half-life of thermal inactivation was calculated using the method as described by Zhao and Arnold [16].

#### pH optimum and pH stability

The optimal pH for lipase activity was determined by incubating the lipase with substrate in a suitable buffer at various pH ranging from 4 to 9, and the maximum lipase activity was considered 100 %. The corresponding buffers were NaAc/HAc(pH4.0-5.0), Na2HPO4-NaH2PO4 (pH6.0-8.0), and Gly/NaOH (pH9.0), respectively, and the concentrations of all used buffers were 20 mmol/L. To determine the effect of pH on lipase stability at pH ranging from 6.0 to 8.5, aliquots of the concentrated LipC24 preparation were diluted five-fold in the corresponding buffer (pH 6.0, pH6.5, pH7.0, pH7.5, pH8.0 and pH8.5) and then incubated for 24 h at 4 °C. The residual lipase activity after incubation was determined and the lipase activity at the start was taken as 100 %.

#### Substrate specificity

The activities of LipC24 toward various 4-nitrophenyl fatty acid esters with varying chain length (C4, C8, C10, C12, C14 and C16) were investigated.

Kinetic parameters for hydrolysis of 4-nitrophenyl myristate (*p*NPM) The Michaelis-Menten constant (*K*_m_) and maximal reaction rate (*V*_max_) of LipC24 hydrolysis activity were determined at different *p*NPM concentration (0.1, 0.2, 0.4, 0.6, 0.8, 1.0, 1.2, 1.4, and 1.8 mmol/L, respectively) under identical conditions to the spectrophotometric assay. Data points were fitted by non-linear regression using Graphpad Prism6.

#### Positional specificity assay

Positional specificity was determined by analyzing lipolysis products of triolein by thin-layer chromatography (TLC) on silica gel GF254, following the procedure described by Rahman et al. [17]. In brief, the reaction mixtures containing 0.1 mol/L of triolein, 1.3 mL Na_2_HPO_4_-NaH_2_PO_4_ (20 mmol/L, pH7.4), and 5 U(500 μL) LipC24 solution were shaken at 200 rpm at 40 °C for 3 h. The reaction products were extracted with *n*-hexane and then analyzed by TLC. The silica gel plate was developed in a mixture of chloroform and acetone (96:4).

The lipase activity of the purified LipC24 was measured using spectrophotometric assay under standard assay conditions, as described by Kordel et al. [18]. The spectrophotometric assay method was used in the whole experiment unless stated otherwise. All reactions were carried out at 40 °C and 20 mmol/L of Na_2_HPO_4_-NaH_2_PO_4_ buffer (pH7.5). One unit of lipase activity was defined as the amount of LipC24 that liberated 1 μmol 4-nitrophenol from 4-nitrophenyl fatty acid ester per min.

### 3D model of LipC24

Three dimensional (3D) structural model of LipC24 was generated and optimized using the software YASARA (version18.4.30; www.yasara.org) with default settings [19].

### Site-directed mutagenesis of the *lipC24* gene

Amino acid substitutions (Ser^179^Ala) in LipC24 were performed using the Quickchange site-directed mutagenesis method. The plasmid pEDSF-*lipB*-*lipC24* was used as template and the complementary mutagenic oligonucleotide primers were listed in Table 2. PCR products were firstly hydrolyzed using *Dpn*I restriction endonuclease to remove methylated parental template DNA, and then transformed into *E. coli* Origami2 (DE3). The desired nucleotide substitutions was confirmed by DNA sequencing.

Induction expression of *lipC24-Ser*^*179*^*Ala* and purification of LipC24-Ser^179^Ala was carried out using the same method as described above.

### Construction of the *lipA*-inactivation mutation strain and the *lipC24-*inactivation mutation strain

To rapidly inactivate *lipA* gene or *lipC24* gene of *Burkholderia* sp. ZYB002, the suicide plasmid pBCMB-S3 (for *lipA*-inactivation) and pBCMB-S5 (for *lipC24*- inactivation) were constructed, respectively. Construction of plasmid pBCMB-S3 and plasmid pBCMB-S5 was adopted the identical method and flow diagram (Additional file 2: Figure S1 and Figure S2). Details of the construction of the *lipA*-inactivation mutation strain was only given.

The suicide plasmid pBCMB-S3 was constructed as follows. Firstly, the full length of the trimethoprim resistance gene was cloned from plasmid pBBR1TP using tmpF/tmpR primers incorporated in the *Bgl* II restriction site. The PCR product was digested with *Bgl* II, and then ligated into the *Bgl* II-digested plasmid pJQ200SK. The resulting plasmid was designated pBCMB-S1. Secondly, the fragment of the *lip*A gene was amplified using lipAMF/lipAMR primers, which were designed to add a *Bam*H I site and a *Xho* I site at the 5’-terminal and the 3’-terminal of the *lip*A gene fragment, respectively. The PCR product (named as *lipA’*) was digested with the respective enzymes and then ligated to the *Xho* I/*Bam*H I-digested plasmid pBCMB-S1. The resulting plasmid was designated pBCMB-S2. Thirdly, the *gfp* gene was cloned from plasmid pEGFP-N1 using gfpF/gfpR primers incorporated the *Pst* I restriction site. The PCR product was digested with *Pst* I, and then ligated into the *Pst* I-digested plasmid pBCMB-S2. The resulting plasmid was designated pBCMB-S3.

The suicide plasmid pBCMB-S3 was delivered to *Burkholderia* sp. ZYB002 by triparental mating as described previously [20]. Candidate mutants were primarily selected on trimethoprim Luria–Bertani (LB) medium and identified by PCR, and further confirmed by Southern blot hybridization using the *gfp* gene fragment labeled with digoxigenin as a probe. The corresponding mutant strain was designated *Burkholderis* sp. ZYB002-Δ*lipA.*

### Cell-bound lipase production and activity assay

Cell-bound lipase production was carried out as described by Shu et al. [11]. Cell-bound lipase activity was determined using alkali titration method as described by Saxena et al. [21]. All reactions were carried out at 40 °C and 20 mmol/L Na_2_HPO_4_-NaH_2_PO_4_ buffer (pH7.5) unless stated otherwise. One unit of cell-bound lipase activity was defined as 1 μmol of fatty acid produced from olive oil per min by the cell culture of 1 OD_600_ under the standard assay conditions.

## Results

### Sequence of LipA, LipC21 and LipC24

**Fig. 1:**
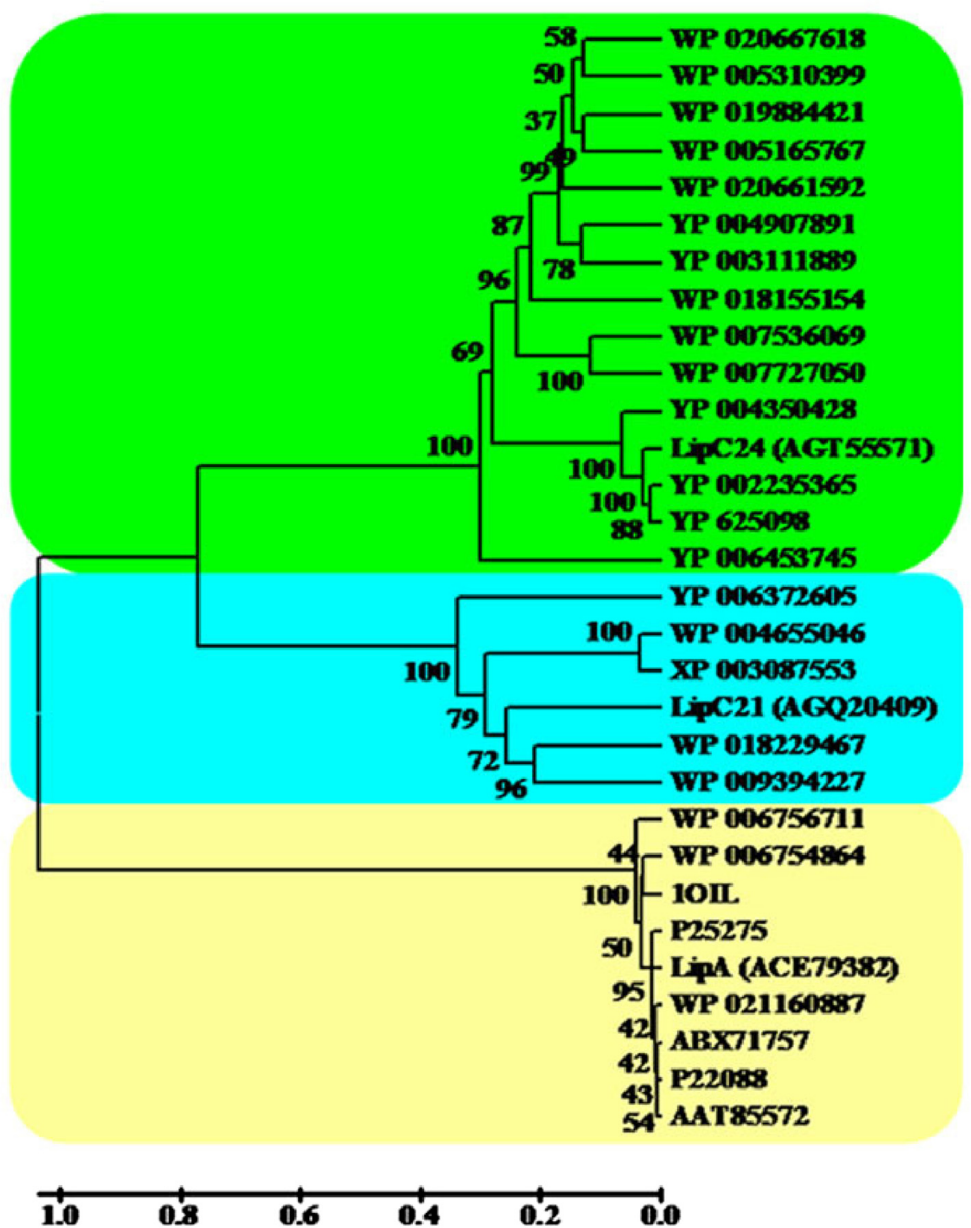
Phylogenetic tree of LipA cluster, LipC21 cluster and LipC24 cluster. The amino acid sequences included the putative, uncharacterized lipases showing over 30 % identity to LipA, LipC21 and LipC24

Nucleotide sequences of the *lipA/B*, *lipC21* and *lipC24* have been deposited in the GenBank database under the accession No. EU768869, No. KF192626, No. KF438175, respectively. Nucleotide sequence analysis revealed that the *lipA* ORF, *lipC21* ORF and *lipC24* ORF coded for a putative protein of 364 amino acids, 427 amino acids and 438 amino acids, respectively. Protein sequence analysis revealed that the LipA displayed 96 % identity with the known lipase from *Pseudomonas* sp. KWI-56 [22], while all homologous protein sequences of LipC21 and LipC24 were the putative and uncharacterized lipases from the whole genomic DNA sequences. Moreover, protein sequence alignment through BLAST did not reveal any sequence identity among LipA, LipC21 and LipC24 (Fig. 1).

### Expression and enzymatic characterization of LipA, LipC21 and LipC24

**Fig. 2:**
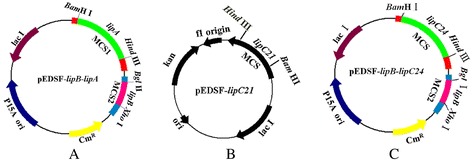
Map of the expression plasmid for *lipA*, *lipC21* and *lipC24*. **a** pEDSF-*lipB*- *lipA* was derived from pACYCDuet-1, which was inserted *lipA* gene at the MCS1 site and the chaperone *lipB* gene at the MCS2 site; **b** pEDSF-*lipC21* was derived from pET28a, which was inserted *lipC21* gene at the MCS site. To obtain the soluble expression of *lipC21*, plasmid pEDSF-*lipC21* and plasmid pGro7 must be co-transformed into *E. coli* BL21(DE3); **c** pEDSF-*lipB*-*lipC24* was derived from pACYCDuet-1, which was inserted *lipC24* gene at the MCS1 site and the chaperone *lipB* gene at the MCS2 site

Soluble LipA, LipC21 and LipC24 could be obtained only when *lipA, lipC21* and *lipC24* were co-expressed with their corresponding chaperone genes (Fig. 2). The soluble expression of *lipA* and *lipC24* required the assistance of the lipase-specific folding gene, *lipB*. However, co-expression of the chaperone *GroEL-GroES* gene is the prerequisite for the soluble expression of *lipC21*. Moreover, the soluble expression level of LipC24 could be significantly increased when *E. coli* Origami2 (DE3) was used as the expression host strain (Data not shown).

**Fig. 3:**
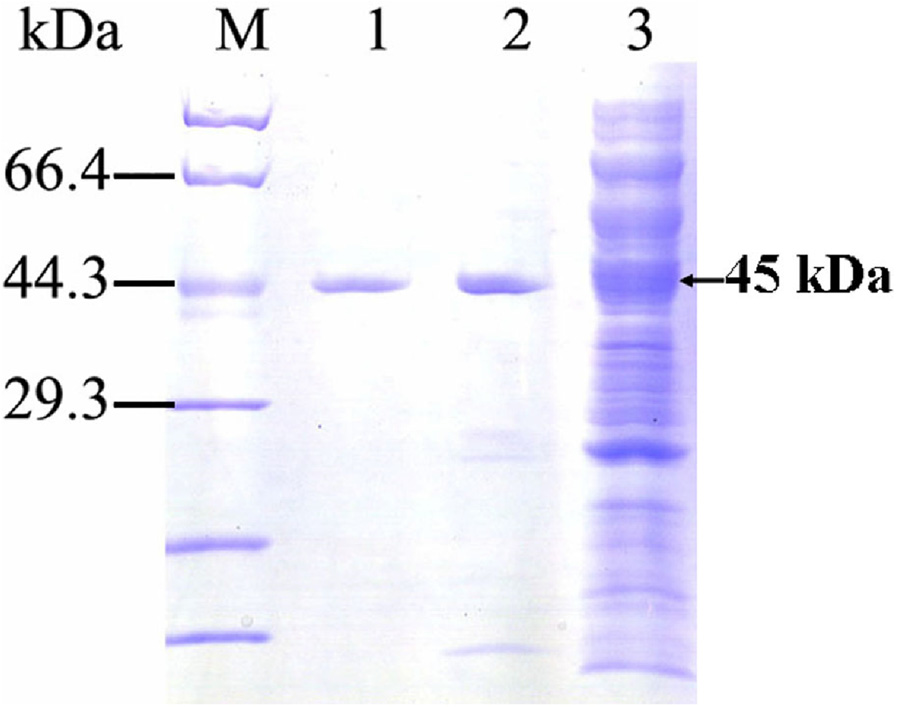
SDS-PAGE analysis of LipC24 in different purification steps. M: protein marker; 1: the purified LipC24 by HiTrap DEAE FF anion-exchange chromatography column; 2: the purified LipC24 by HisTrap HP affinity chromatography column; 3: cell-free extract of *E. coli* Origami 2(DE3)-pEDSF-*lipB*-*lipC24*

**Fig. 4:**
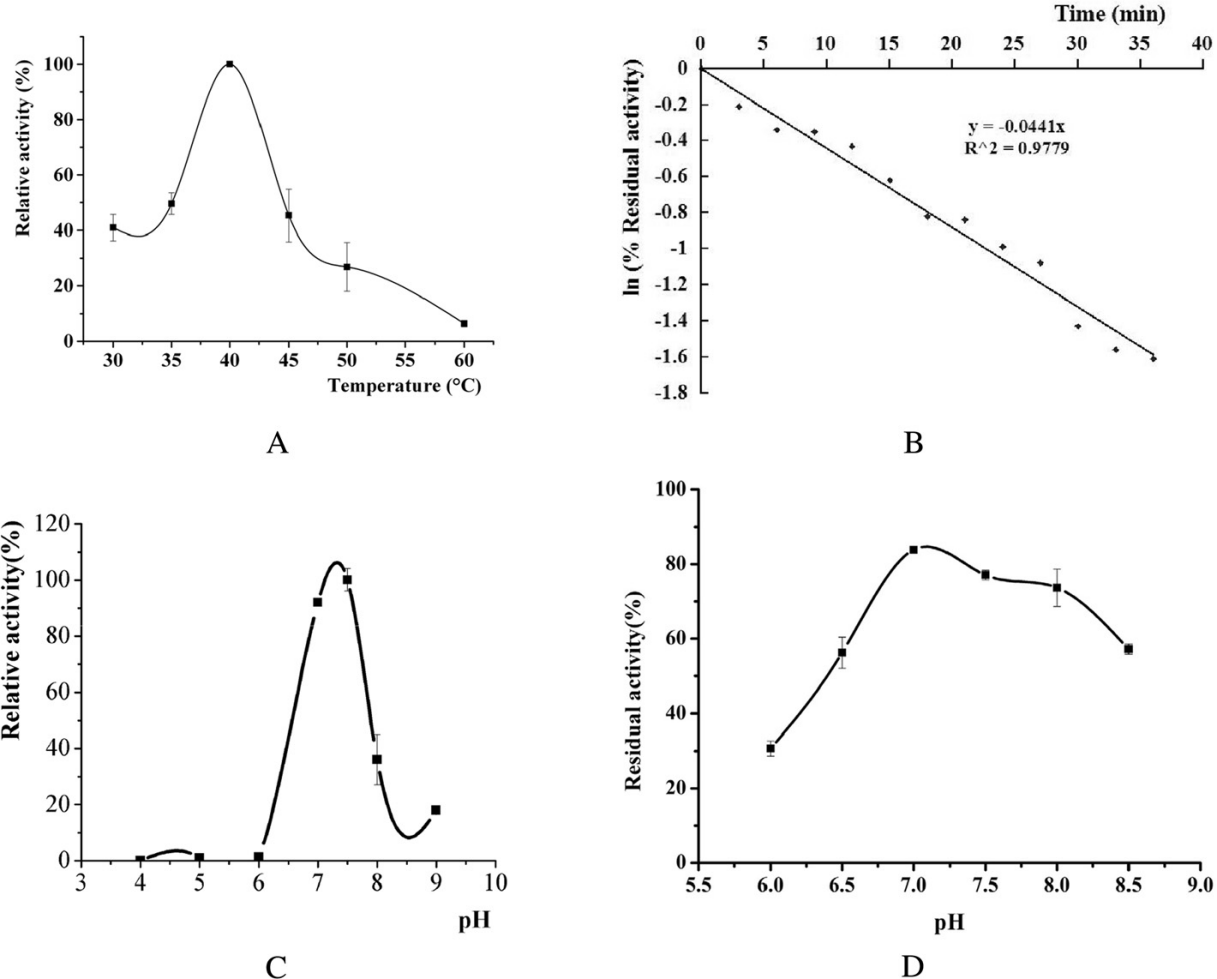
Enzymatic characterization of the purified LipC24. **a** Effect of temperature on LipC24 activity; **b** Effect of temperature on LipC24 stability; **c** Effect of pH on LipC24 activity; **d** Effect of pH on LipC24 stability

**Fig. 5:**
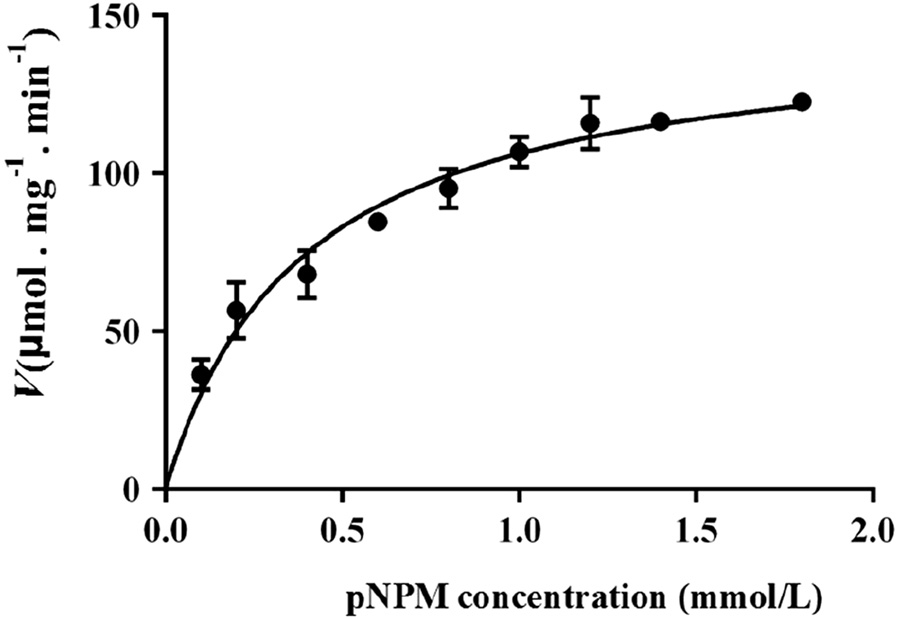
Kinetic plot of 4-nitrophenyl myristate hydrolysis catalyzed by LipC24

**Fig. 6:**
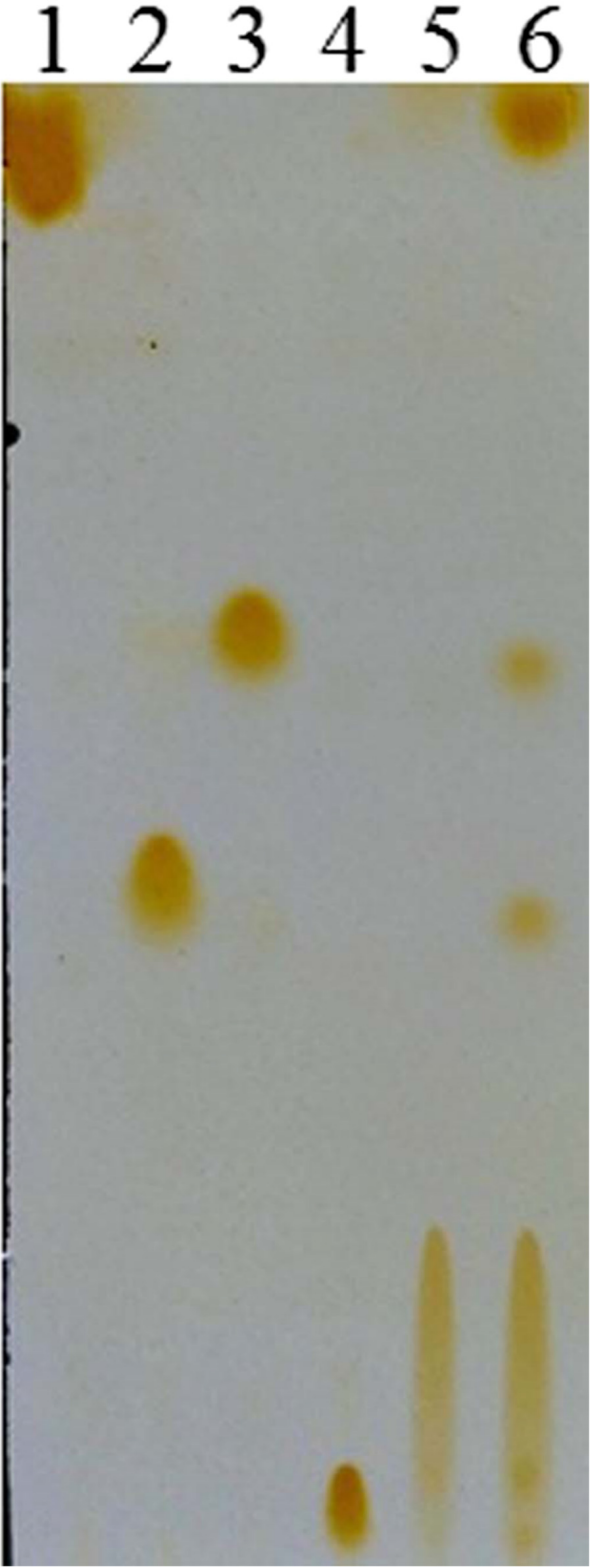
Thin-layer chromatogram of the hydrolysis products of triolein catalyzed by LipC24. Lane 1, Triolein; Lane 2, 1, 2-Diolein; Lane 3, 1, 3-Diolein; Lane 4, 1-Monoolein; Lane 5, Oleic acid; Lane 6, hydrolysis products of triolein

Except for LipC21, both LipA and LipC24 displayed lipase activity. As most reports on the enzymatic characterization of the LipA from *B. cepacia*, the relative molecular weight of the LipA from *Burkholderia* sp. ZYB002 was 34 kDa. LipA was an alkaline mesothermal-active lipase [23]. The optimum temperature and pH of LipA for hydrolysis activity were 40 °C and 8.0, respectively [23]. The enzymatic characterization of LipC24 was totally different from that of LipA. The LipC24 was purified 17.7-fold from the supernatant of the *E. coli* cell lysate and yielded 21.49 % of the initial activity. The specific activity of LipC24 was 15.63 U/mg using 4-nitrophenyl palmitate as substrate (Table 3), which was far lower than that of LipA (253.82 U/mg for 4-nitrophenyl palmitate) [23]. SDS/PAGE analysis of LipC24 displayed a single band, which corresponded to a molecular mass of 45 kDa (Fig. 3). The optimum temperature and pH of LipC24 for hydrolysis activity were found to be 40 °C and 7.5, respectively. The LipC24 could be kept stable in the pH range 7.0-8.0 for 24 h at 4 °C, while the half-time of the LipC24 was only 16 min at 40 °C (Fig. 4). LipC24 was less stable than LipA. LipA displayed excellent thermostability up to 65 °C and could keep stability over a broad pH range from 3.0 to 10 [23]. The LipC24 indicated a clear preference for esters with the medium acyl chain length (C10-C14) when assayed using 4-nitrophenyl derivatives (Table 4). The LipC24 exhibited a simple Michaelis-Menten kinetics for *p*NPM hydrolysis. The values of *K*_m_ and *V*_max_ of LipC24 were 0.37 ± 0.07 mmol/L and 138.8 ± 7.90 μmol **·** min^−1^ **·** mg^−1^, respectively (Fig. 5). Michaelis constan *K*_*m*_ of LipC24 is less than that of lipase from *Bacillus* sp.. Accordingly, the maximum reaction velocity *V*_max_ of LipC24 was higher than that of *Bacillus* sp. [24]. LipC24 cleaved not only the 3-positioned ester bonds, but also the 2-positioned ester bond of triolein (Fig. 6). Thus, LipC24 could nonspecifically hydrolyze the ester bonds of triolein. The same experiment results were verified with other *Pseudomonas* sp. lipases [17, 25].

**Table 3:** Purification of LipC24 from *E. coli* Origami 2(DE3)-pEDSF-*lipB*-*lipC24*

Steps	Total activity (U)	Total protein (mg)	Specific activity (U/mg)	Yield (%)	Purification (fold)
Cell-free extract	245.39	89.78	2.73	100	1
HisTrap HP	57.96	1.8	32.20	23.62	11.79
HiTrap DEAE FF	52.74	1.09	48.31	21.49	17.70

**Table 4:** The specific activity of LipC24 towards 4-nitrophenyl esters

Substrate	Specific activity (U/mg)
4-nitrophenyl palmitate (C16)	15.63 ± 1.08
4-nitrophenyl myristate (C14)	55.49 ± 1.87
4-nitrophenyl laurate (C12)	31.14 ± 2.59
4-nitrophenyl decanoate (C10)	48.31 ± 2.06
4-nitrophenyl octanoate (C8)	18.54 ± 1.67
4-nitrophenyl butyrate (C4)	0.51 ± 0.12

### Protein sequence and structural model analysis of LipC24

**Fig. 7:**
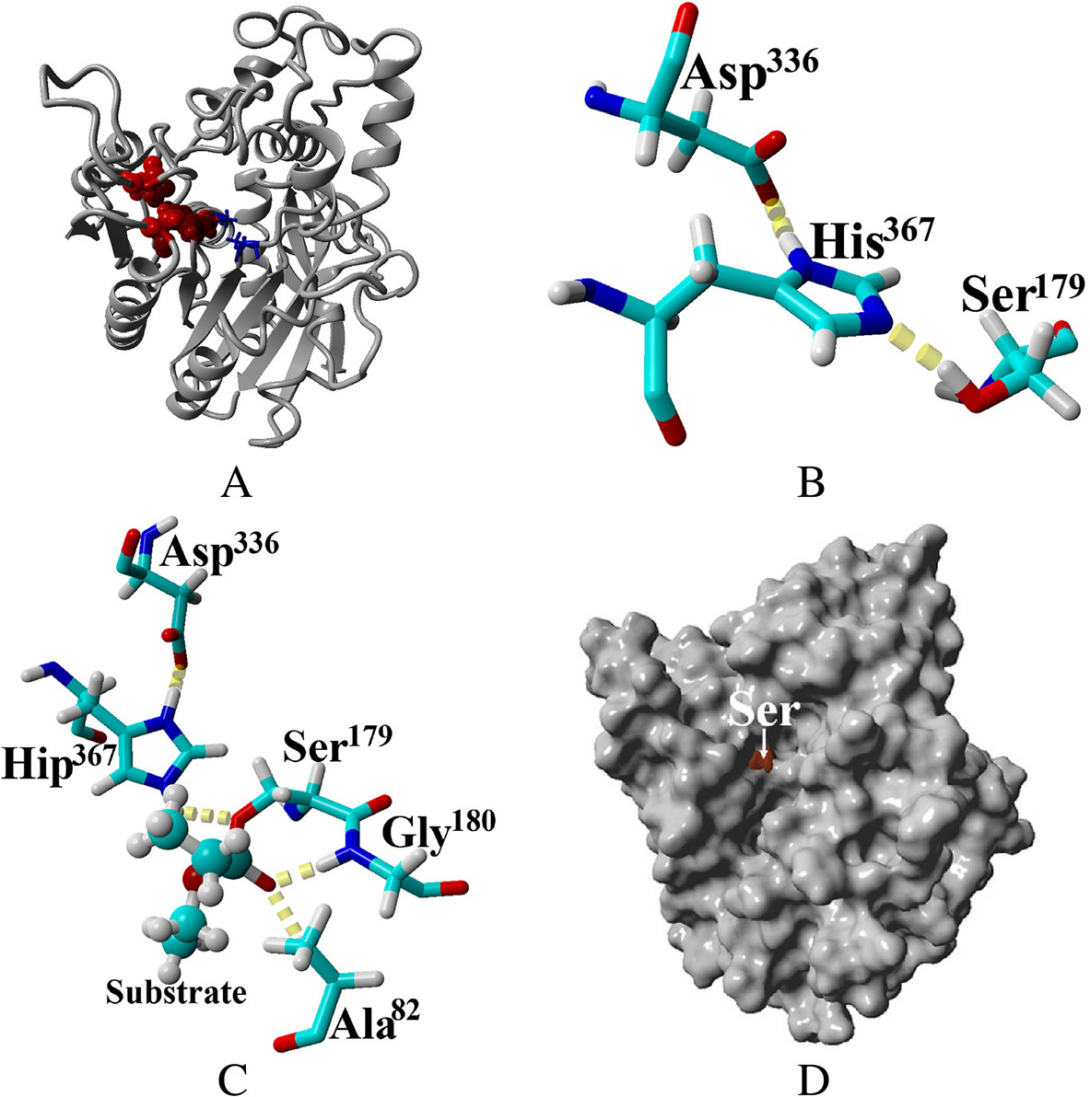
The 3D model of LipC24. **a** The overall three-dimensional structure of LipC24. β-strands were represented as arrows and surrounded by the helices; **b** Ser^179^, Asp^336^, and His^367^ formed the catalytic triad within the range of H-bond interactions; **c** The transient state model of LipC24-ethyl acetate complex, which was stabilized by Ala^82^ and Gly^180^. Hip^367^ originated from His^367^, which accepted a proton from the hydroxyl group of Ser^179^; **d** The open Y-type substrate-binding pocket of LipC24

There were several conserved sequence blocks between the deduced amino acid sequence of LipC24 and the other putative homologous lipases. In block 3, there was a conserved pentapeptide Gly-Tyr-Ser-Gly-Gly, in which the catalytic serine residue was embedded in most lipases. Besides the conserved serine residue, there were three conserved aspartate residues in block 1, block 2 and block 5, and a conserved histidine residue in block 5 (Additional file 2: Figure S3). The 3D homology model of LipC24 presented the characteristics of a canonical α/β-hydrolase fold, in which parallel or mixed β sheet in the molecular center was surrounded (or connected) by helices (Fig. 7a). A hydrogen bond network was formed among Ser^179^, Asp^336^, and His^367^, which constituted the catalytic triad (Fig. 7b). Mutant of LipC24-Ser^179^Ala lost 100 % lipase activity, which confirmed the function of Ser^179^ in the active site. The oxyanion hole consisted of Ala^82^ and Gly^180^, which stabilized the transient state of LipC24-ethyl acetate complex (Fig. 7c). The substrate-binding pocket of LipC24 displayed the distinct open Y-type structure (Fig. 7d).

### Component of the cell-bound lipase

**Fig. 8:**
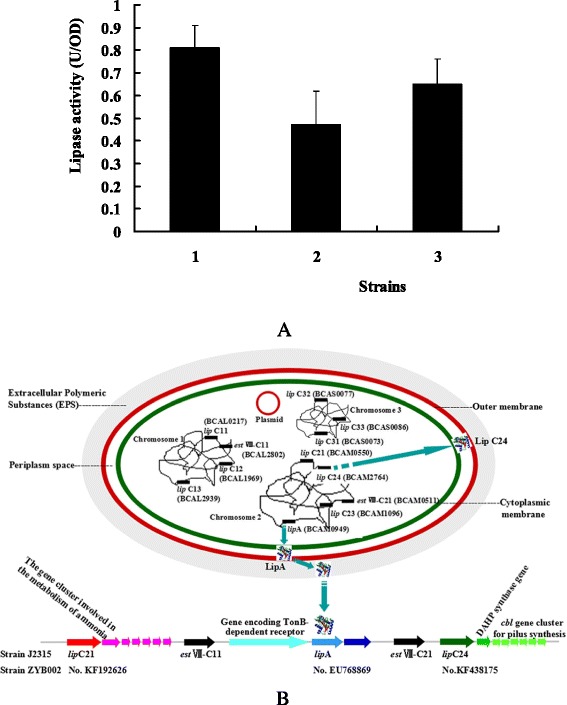
Component of the cell-bound lipase from *Burkholderia* sp. ZYB002. **a** Comparsion analysis of the cell-bound lipase activity from *Burkholderis* sp. ZYB002, *Burkholderis* sp. ZYB002-Δ*lipA*, and *Burkholderis* sp. ZYB002-Δ*lipC24*. 1 *Burkholderis* sp. ZYB002 strain; 2 *Burkholderis* sp. ZYB002-Δ*lipA* strain; 3 *Burkholderis* sp. ZYB002-Δ*lipC24* strain. **b** The predictive lipase gene family and two family VIII esterase genes (*estVIII-C11* and *estVIII-C21*) from *B.cepacia* J2315

The cell-bound lipase activity of *Burkholderis* sp. ZYB002-Δ*lipA* and *Burkholderis* sp. ZYB002-Δ*lipC24* significantly decreased to 58 % and 86 % of its original activity, respectively (Fig. 8a)*.* The cell-bound lipase activity originated from a multi-enzyme mixture in which LipA was the main component. Besides LipA and LipC24, other type of lipases could exist on the cell surface of *Burkholderis* sp. ZYB002.

## Discussion

Different types of lipase produced by a specific microbial strain always displayed a totally different 3D structure and enzymatic characterization [26–28]. In previous research, cell-bound lipase from *B. cepacia* displayed excellent catalytic activity for organic synthesis [12, 13]. However, there was not any report on the gene sequence nor any structural investigation of the cell-bound lipase genes from *B. cepacia.* From the whole genomic DNA sequence of *B. cepacia* J2315 (www.burkholderia.com), more than 10 gene sequences were predicted as lipase genes, including the extracellular lipase *lipA* [gene locus: BCAM0949] and the chaperone gene *lipB* [gene locus: BCAM0949]. Moreover, two predictive lipases, LipC21 [gene locus: BCAL1969] and LipC24 [gene locus: BCAM2764], were distributed on the cytoplasmic membrane. The genes, *lipC21* and *lipC24* were situated on the gene cluster for ammonia metabolism and pilus synthesis, respectively (Fig. 8b). In *P. aeruginosa*, part of the extracellular lipase could be anchored on the cell surface and act as cell-bound lipase [4–6].

Three speculative cell-bound lipase genes (*lipA*, *lipC21* and *lipC24*) from *Burkholderia* sp. ZYB002 were cloned and expressed in *E. coli*. Among LipA, LipC21 and LipC24, only LipA displayed a high sequence identity with the known extracellular lipase from *Pseudomonas* sp. KWI-56 [22], which suggested that LipA was the authentic triacylglycerol lipase. The protein sequences of LipC21 and LipC24 had not any sequence identity with known lipases or esterases, which could lead to speculations that LipC21 and LipC24 were the novel lipases (Fig. 1).

The expression soluble lipases required different chaperone protein genes. It was necessary for *lipA* and *lipC24* to be co-expressed with the *lipB* gene. Due to the strong hydrophobicity, a 70-amino acid residue fragment at the N-terminal of LipB had to be truncated when *lipB* was heterogeneously co-expressed in *E. coli* [29]. Among *lipB*, *groES-groEL* gene, *dnaK-dnaJ-grpE* gene and *tig* gene, it was only the chaperone *groES-groEL* gene that improved the soluble expression level of *lipC21. GroEL-GroES* was also reported to be necessary for the soluble expression of the family VIII lipase *lipBL* from *Marinobacter lipolyticus* [15].

Enzymatic characterization and 3D structure of LipC24 was totally different from that of LipA. LipC24 displayed high activity in the neutral buffer (pH7.0-7.5) and mesothermal reaction conditions. Furthermore, LipC24 would sharply abolish the lipolytic activity when LipC24 was kept at high temperature, alkaline solution, or acid solution, respectively. On the contrary, LipA was thermostable, alkaline-tolerant, and organic solvent-resistant [30, 31]. The open Y-type active site of LipC24 was totally different from the funnel-shaped active site of LipA [32]. In the molecular model of LipC24, a predictive intramolecular disulfide bond was formed between Cys^352^ and Cys^395^ (PredictProtein 2013 server, https://www.predictprotein.org/), which corresponded to the requirement of the host strain, *E. coli* Origami2 (DE3) for the soluble expression of *lipC24*.

Although both titrimetric assay method and colorimetric assay method were widely used for lipase activity determination, there were obvious differences between the two assay methods [33, 34]. Olive oil or other triacylglycerol was used as the substrate in the titrimetric assay method, while 4-nitrophenyl esters were always used as the substrate in the colorimetric assay method. However, 4-nitrophenyl esters could be permeated into the cytoplasm [35–37], and was hydrolyzed by the intracellular lipolytic enzymes (including lipase and esterase). 4-nitrophenyl esters could not be used as the substrates for activity determination of the cell-bound lipase. In the present work, membrane-impermeable olive oil and the alkali titration assay method was used for the activity determination of the whole cell lipase.

The cell-bound lipase activity of *Burkholderis* sp. ZYB002-Δ*lipA* decreased by 42 % of the total cell-bound lipase activity *Burkholderis* sp. ZYB002 (Fig. 8a), which demonstrated that LipA was the main component of cell-bound lipases. It had been reported that microbial strains could simultaneously produce extracellular lipases and various kinds of biosurfactants (rhamnolipid, lipopolysaccharide, polysaccharide alginate, etc.) when various oils or lipids were used as the inducer or carbon source [4, 38]. Part of biosurfactants were firmly associated with the outer membrane of the host strain and could interact with lipases by electrostatic interaction, which resulted in cell surface anchoring of the extracellular lipases [5, 6]. LipC24 contributed 14 % of the total cell-bound lipase activity *Burkholderis* sp. ZYB002 (Fig. 8a). Besides LipA and LipC24, other type of the cell-bound lipases (or esterases) could exist. Further analysis of the whole genome DNA sequence of *Burkholderia cepacia* J2315 predicted several esterase gene sequences, including two novel family VIII esterase genes, *estVIII-C11* [gene locus: BCAL2802] and *estVIII-C21* [gene locus: BCAM0511] (Fig. 8b).

## Conclusions

The cell-bound lipase activity of *Burkholderia* sp. ZYB002 was shown to be a multi-enzyme mixture, which at least consisted of LipA and LipC24. LipA was the main component of the cell-bound lipase. LipC24 was a novel lipase, which displayed a totally different enzymatic characterization and 3D structure to that of LipA. Besides LipA and LipC24, other type of the cell-bound lipases (or esterases) may exist.

### Ethics approval and consent to participate

Not applicable.

### Consent for publication

Not applicable.

### Availability of data and materials

The datasets supporting the conclusions of this article are included within the article and its additional files.

## Additional files

Additional file 1: All PCR conditions and PCR procedures used in this research. (DOC 54 kb) Additional file 2: Figure S1. The construction flow diagram for the suicide plasmid pBCMB-S3, which was used to construct the *lipA*-inactivation mutation strain. Figure S2. The construction flow diagram for the suicide plasmid pBCMB-S5, which was used to construct the *lipC24*-inactivation mutation strain. Figure S3. Blocks of sequences conserved between LipC24 and other putative homologous lipases. (DOC 1407 kb)

## Abbreviations

*gfp*: green fluorescent protein-encoding gene
IPTG: isopropyl-beta-d-thiogalactopyranoside
pNPM: 4-nitrophenyl myristate
SDS-PAGE: sodium lauryl sulfate-polyacrylamide gel electrophoresis
TLC: thin-layer chromatography
