# Supplementary material for: Cell-bound lipases from Burkholderia sp. ZYB002: gene sequence analysis, expression, enzymatic characterization, and 3D structural model
Source: BMC Biotechnol. 2016 May 3;16:38. doi: 10.1186/s12896-016-0269-6 (PMC4855798; doi:10.1186/s12896-016-0269-6)
Supplement: Additional file 1: — All PCR conditions and PCR procedures used in this research. (DOC 54 kb) [file 12896_2016_269_MOESM1_ESM.doc]

PCR conditions and PCR procedures used in this research

1. PCR amplification for the full length of *lipA*/*lipB*

A 20 µL reaction contained 0.4 mmol/L dATP, dGTP, dCTP and dTTP, 1 U Taq DNA polymerase, 1 µmol/L of each primer (lipACF and lipACR, Table 2) and 0.1 µg of genomic DNA from *Burkholderia* sp. ZYB002. The PCR program comprised 5 min at 94°C followed by 25 cycles of 94°C for 1 min, 52°C for 1 min, and 72°C for 3 min with a final extension of 72°C for 7 min. The PCR product was cloned into pMD18T, which resulted in plasmid of pMD18T-*lipAB.*

2. PCR amplification for the full length of *lipC21*

A 20 µL reaction contained 0.3 mmol/L dATP, dGTP, dCTP and dTTP, 1 U Taq DNA polymerase, 1 µmol/L of each primer (lipC21CF and lipC21CR, Table 2) and 0.1 µg of genomic DNA from *Burkholderia* sp. ZYB002. The PCR program comprised 5 min at 94°C followed by 25 cycles of 94°C for 30 sec, 60°C for 30 sec, and 72°C for 1 min with a final extension of 72°C for 7 min. The PCR product was cloned into pMD18T, which resulted in the plasmid of pMD18T-*lipC21*

3. PCR amplification for the full length of *lipC24*

A 20 µL reaction contained 0.25 mmol/L dATP, dGTP, dCTP and dTTP, 1 U Taq DNA polymerase, 1 µmol/L of each primer (lipC24CF and lipC24CR, Table 2) and 0.1 µg of genomic DNA from *Burkholderia* sp. ZYB002. The PCR program comprised 5 min at 94°C followed by 25 cycles of 94°C for 30 sec, 60°C for 30 sec, and 72°C for 1 min with a final extension of 72°C for 7 min. The PCR product was cloned into pMD18T, which resulted in the plasmid of pMD18T-*lipC24.*

4. PCR amplification for the fragment of *lipB*

A 20 µL reaction contained 0.25 mmol/L dATP, dGTP, dCTP and dTTP, 1 U Taq DNA polymerase, 1 µmol/L of each primer (lipBEF and lipBER, Table 2) and 0.1 µg of genomic DNA from *Burkholderia* sp. ZYB002. The PCR program comprised 5 min at 94°C followed by 25 cycles of 94°C for 30 sec, 75°C for 20 sec, and 72°C for 1 min with a final extension of 72°C for 7 min. The PCR product was digested with *Bgl* II/*Xho* I and then ligated into the *Bgl* II/*Xho* I-digested plasmid pACYCDuet-1. The resulting plasmid was designated pEDSF-*lipB*.

5. PCR amplification for the coding region of the mature polypeptide LipA

A 20 µL reaction contained 0.25 mmol/L dATP, dGTP, dCTP and dTTP, 1 U Taq DNA polymerase, 1 µmol/L of each primer (lipAEF and lipAER, Table 2) and 50 ng of plasmid pMD18T-*lipAB*. The PCR program comprised 5 min at 94°C followed by 25 cycles of 94°C for 30 sec, 73°C for 20 sec, and 72°C for 1 min with a final extension of 72°C for 7 min. The PCR product was digested with *Bam*H I/*Hin*d III and then ligated into the *Bam*H I/*Hin*d III-digested plasmid pEDSF-*lipB*. The resulting plasmid was designated pEDSF-*lipB*-*lipA.*

6. PCR amplification for the coding region of the mature polypeptide LipC21

A 20 µL reaction contained 0.25 mmol/L dATP, dGTP, dCTP and dTTP, 1 U Taq DNA polymerase, 1 µmol/L of each primer (lipC21EF and lipC21ER, Table 2) and 60 ng of plasmid pMD18T-*lipC21.* The PCR program comprised 5 min at 94°C followed by 25 cycles of 94°C for 30 sec, 60°C for 30 sec, and 72°C for 1 min with a final extension of 72°C for 7 min. The PCR was digested with *Bam*H I/*Hin*d III and then ligated into the *Bam*H I/*Hin*d III-digested plasmid pET28a. The resulting plasmid was designated pEDSF-*lipC21.*

7. PCR amplification for the coding region of the mature polypeptide LipC24

A 20 µL reaction contained 0.25 mmol/L dATP, dGTP, dCTP and dTTP, 1 U Taq DNA polymerase, 1 µmol/L of each primer (lipC24EF and lipC24ER, Table 2) and 60 ng of plasmid pMD18T-*lipC24*. The PCR program comprised 5 min at 94°C followed by 25 cycles of 94°C for 30 sec, 60°C for 30 sec, and 72°C for 1 min with a final extension of 72°C for 7 min. The PCR was digested with *Bam*H I/*Hin*d III and then ligated into the *Bam*H I/*Hin*d III-digested plasmid pEDSF-*lipB*. The resulting plasmid was designated pEDSF-*lipB*-*lipC24.*

8. PCR amplification to introduce a mutation in the gene, *lipC24*, for the substitution Ala for Ser^179^

A 20 µL reaction contained 0.25 mmol/L dATP, dGTP, dCTP and dTTP, 1 U Taq DNA polymerase, 1 µmol/L of each primer (lipC24MF and lipC24MR, Table 2) and 60 ng of plasmid pEDSF-*lipB*-*lipC24*. The PCR program comprised 5 min at 94°C followed by 25 cycles of 94°C for 30 sec, 60°C for 30 sec, and 72°C for 6 min with a final extension of 72°C for 7 min. The PCR product was incubated overnight at 37°C with 0.8 U of *Dpn* I endonuclease in order to digest the template DNA. The residual PCR product was designated pEDSF-*lipB*-*lipC24-Ser^179^Ala.*

9. PCR amplification for the full length of the trimethoprim resistance gene

A 20 µL reaction contained 0.25 mmol/L dATP, dGTP, dCTP and dTTP, 1 U Taq DNA polymerase, 1 µmol/L of each primer (tmpF and tmpR, Table 2) and 50 ng of plasmid pBBR1TP. The PCR program comprised 5 min at 94°C followed by 25 cycles of 94°C for 30 sec, 53°C for 30 sec, and 72°C for 1 min with a final extension of 72°C for 7 min. The PCR product was digested with *Bgl* II, and then ligated into the *Bgl* II-digested plasmid pJQ200SK. The resulting plasmid was designated pBCMB-S1.

10. PCR amplification for the fragment of *lipA*

A 20 µL reaction contained 0.25 mmol/L dATP, dGTP, dCTP and dTTP, 1 U Taq DNA polymerase, 1 µmol/L of each primer (lipAIF and lipAIR, Table 2) and and 80 ng of plasmid pMD18T-*lipAB*. The PCR program comprised 5 min at 94°C followed by 25 cycles of 94°C for 30 sec, 53°C for 30 sec, and 72°C for 1 min with a final extension of 72°C for 7 min. The PCR product was digested with *Bam*H I/*Xho* I and then ligated into the *Bam*H I/*Xho* I-digested plasmid pBCMB-S1. The resulting plasmid was designated pBCMB-S2*.*

11. PCR amplification for the full length of *gfp*

A 20 µL reaction contained 0.25 mmol/L dATP, dGTP, dCTP and dTTP, 1 U Taq DNA polymerase, 1 µmol/L of each primer (gfpF-lipA and gfpR-lipA, Table 2) and 70 ng of plasmid pEGFP-N1. The PCR program comprised 5 min at 94°C followed by 25 cycles of 94°C for 30 sec, 53°C for 30 sec, and 72°C for 1 min with a final extension of 72°C for 7 min. The PCR product was digested with *Pst* I, and then ligated into the *Pst* I-digested plasmid pBCMB-S2. The resulting plasmid was designated pBCMB-S3.

12. PCR amplification for the fragment of *lipC24*

A 20 µL reaction contained 0.25 mmol/L dATP, dGTP, dCTP and dTTP, 1 U Taq DNA polymerase, 1 µmol/L of each primer (lipC24IF and lipC24IR, Table 2) and 60 ng of plasimd pMD18T-*lipC24*. The PCR program comprised 5 min at 94°C followed by 25 cycles of 94°C for 30 sec, 66°C for 30 sec, and 72°C for 1 min with a final extension of 72°C for 7 min. The PCR product was digested with *Apa* I/*Xba* I and then ligated into the *Apa* I/*Xba* I-digested plasmid pBCMB-S1. The resulting plasmid was designated pBCMB-S4*.*

13. PCR amplification for the full length of *gfp*

A 20 µL reaction contained 0.25 mmol/L dATP, dGTP, dCTP and dTTP, 1 U Taq DNA polymerase, 1 µmol/L of each primer (gfpF-lipC24 and gfpR-lipC24, Table 2) and 70 ng of plasmid pEGFP-N1. The PCR program comprised 5 min at 94°C followed by 25 cycles of 94°C for 30 sec, 56°C for 30 sec, and 72°C for 1 min with a final extension of 72°C for 7 min. The PCR product was digested with *Nde* I, and then ligated into the *Nde* I-digested plasmid pBCMB-S4. The resulting plasmid was designated pBCMB-S5.
