# Supplementary material for: Cell-bound lipases from Burkholderia sp. ZYB002: gene sequence analysis, expression, enzymatic characterization, and 3D structural model
Source: BMC Biotechnol. 2016 May 3;16:38. doi: 10.1186/s12896-016-0269-6 (PMC4855798; doi:10.1186/s12896-016-0269-6)
Supplement: Additional file 2: Figure S1. — The construction flow diagram for the suicide plasmid pBCMB-S3, which was used to construct the lipA-inactivation mutation strain. Figure S2. The construction flow diagram for the suicide plasmid pBCMB-S5, which was used to construct the lipC24-inactivation mutation strain. Figure S3. Blocks of sequences conserved between LipC24 and other putative homologous lipases. (DOC 1407 kb) [file 12896_2016_269_MOESM2_ESM.doc]

| **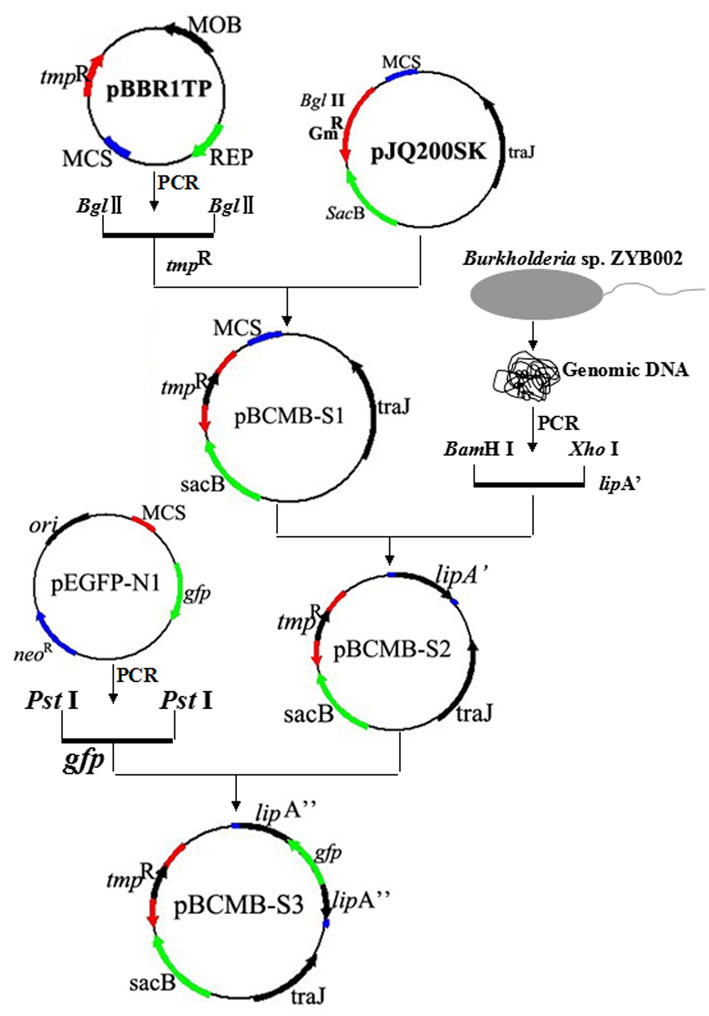** |
| --- |
| Fig. S1 The construction flow diagram for the suicide plasmid pBCMB-S3, which was used to construct the *lipA*-inactivation mutation strain. |

| **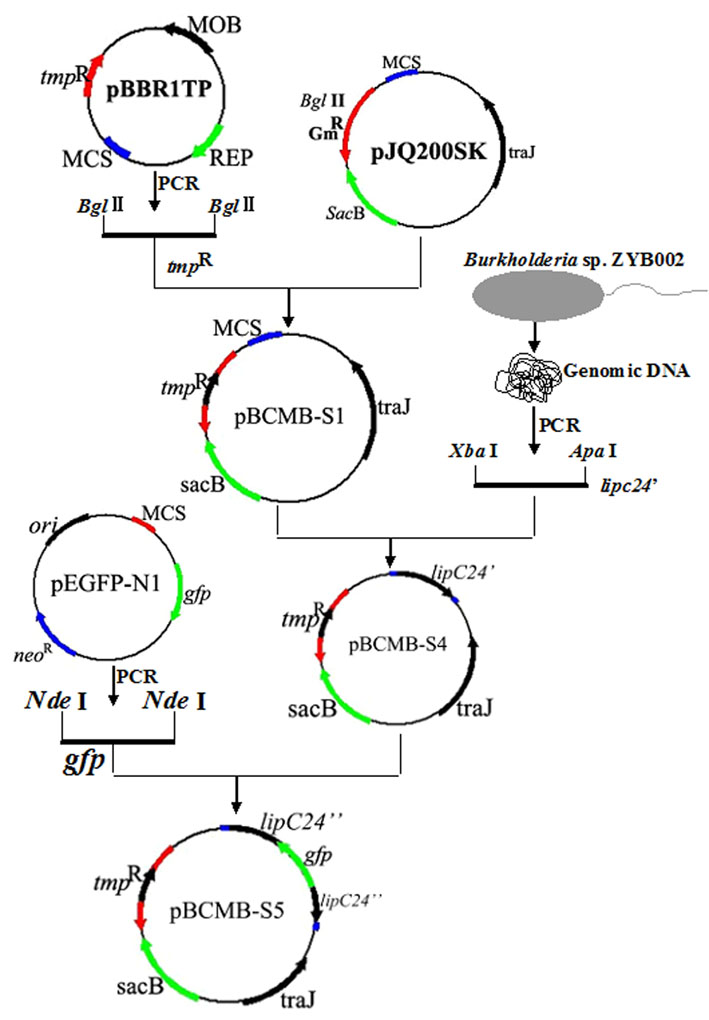** |
| --- |
| Fig. S2 The construction flow diagram for the suicide plasmid pBCMB-S5, which was used to construct the *lipC24*-inactivation mutation strain |

|  |  | Block1 |  | Block2 |
| --- | --- | --- | --- | --- |
| AGT55571(LipC24) | 104 | 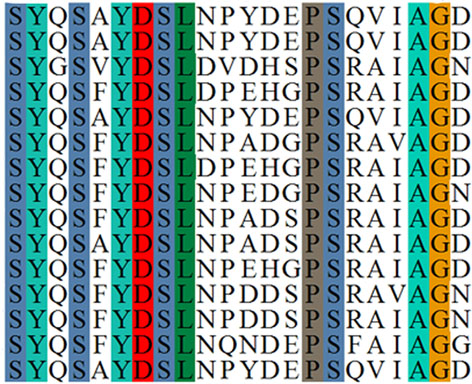 | 168 | 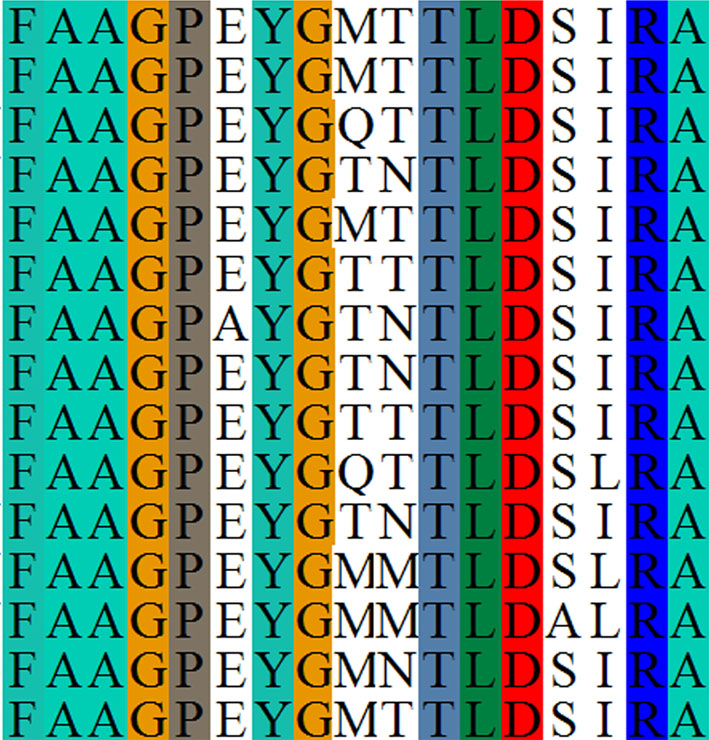 |
| YP_002235365 | 104 |  | 168 |  |
| WP_018155154 | 112 |  | 170 |  |
| WP_020667618 | 114 |  | 172 |  |
| YP_004350428 | 104 |  | 167 |  |
| YP_004907891 | 108 |  | 167 |  |
| WP_005310399 | 116 |  | 174 |  |
| WP_019884421 | 117 |  | 175 |  |
| YP_003111889 | 109 |  | 167 |  |
| WP_020661592 | 102 |  | 160 |  |
| WP_005165767 | 104 |  | 162 |  |
| WP_007536069 | 98 |  | 168 |  |
| WP_007727050 | 110 |  | 180 |  |
| YP_006453745 | 98 |  | 159 |  |
| YP_625098 | 106 |  | 170 |  |
|  |  | Block3 |  | Block4 |
| AGT55571(LipC24) | 203 | 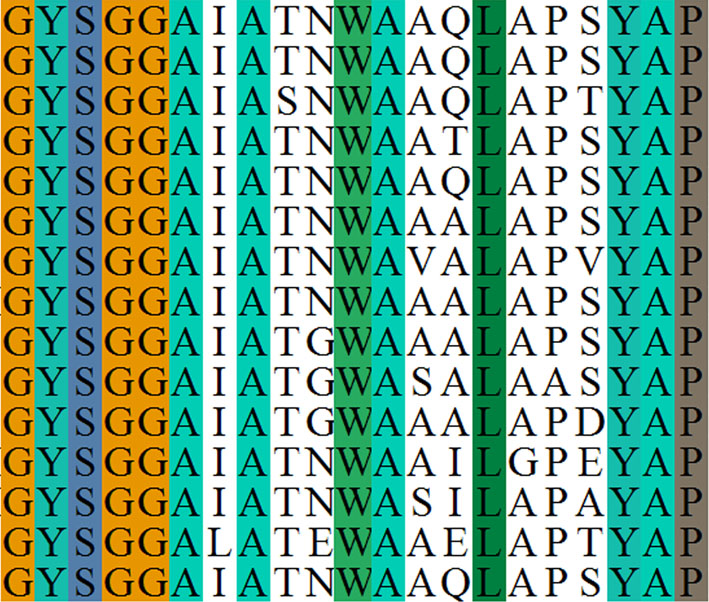 | 235 | 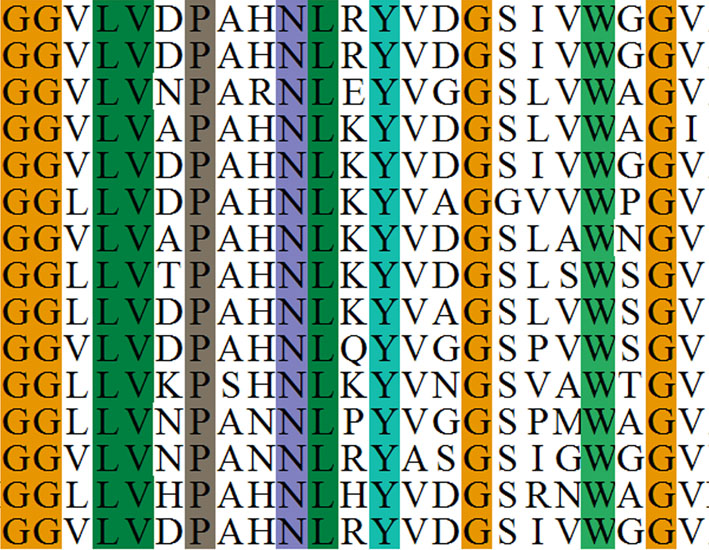 |
| YP_002235365 | 203 |  | 235 |  |
| WP_018155154 | 206 |  | 238 |  |
| WP_020667618 | 207 |  | 239 |  |
| YP_004350428 | 202 |  | 234 |  |
| YP_004907891 | 202 |  | 234 |  |
| WP_005310399 | 209 |  | 241 |  |
| WP_019884421 | 210 |  | 242 |  |
| YP_003111889 | 203 |  | 235 |  |
| WP_020661592 | 195 |  | 227 |  |
| WP_005165767 | 193 |  | 229 |  |
| WP_007536069 | 203 |  | 235 |  |
| WP_007727050 | 215 |  | 247 |  |
| YP_006453745 | 195 |  | 226 |  |
| YP_625098 | 205 |  | 237 |  |
|  |  | Block5 | | |
| AGT55571(LipC24) | 361 | 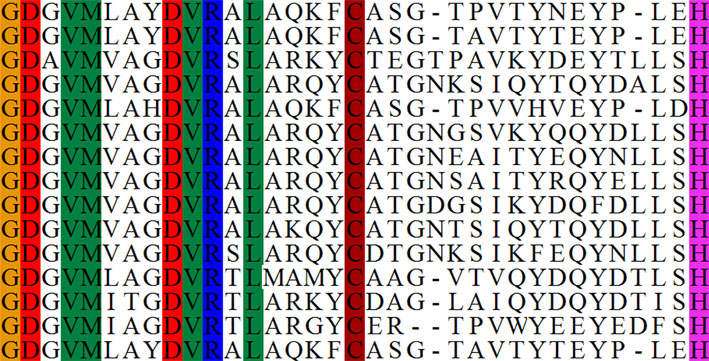 | | |
| YP_002235365 | 361 |  |  |  |
| WP_018155154 | 367 |  |  |  |
| WP_020667618 | 368 |  |  |  |
| YP_004350428 | 360 |  |  |  |
| YP_004907891 | 363 |  |  |  |
| WP_005310399 | 370 |  |  |  |
| WP_019884421 | 371 |  |  |  |
| YP_003111889 | 364 |  |  |  |
| WP_020661592 | 356 |  |  |  |
| WP_005165767 | 358 |  |  |  |
| WP_007536069 | 360 |  |  |  |
| WP_007727050 | 376 |  |  |  |
| YP_006453745 | 366 |  |  |  |
| YP_625098 | 363 |  |  |  |
| Fig. S3 Blocks of sequences conserved between LipC24 and other putative homologous lipases. AGT55571: LipC24 from *Burkholderia* sp. ZYB002; YP_002235365: putative lipase from *B. cenocepacia* J2315; WP_018155154: putative lipase from *D. terragena*; WP_020667618: putative lipase from *A. nigrescens*; YP_004350428: putative lipase from *B. gladioli*; YP_004907891: putative lipase from *K. setae*; WP_005310399: putative lipase from *S. pristinaespiralis*; WP_019884421: putative lipase from *S. purpureus*; YP_003111889: putative lipase from *C. acidiphila* DSM 44928; WP_020661592: putative lipase from *A. benzoatilytica*; WP_005165767: putative lipase from *A. azurea*; WP_007536069: putative lipase from *R. triatomae*; WP_007727050: putative lipase from *R. qingshengii*; YP_006453745: putative lipase from *M. chubuense*; YP_625098: putative lipase from *B. cenocepacia.* | | | | |
